# Ionic switch controls the DNA state in phage λ

**DOI:** 10.1093/nar/gkv611

**Published:** 2015-06-19

**Authors:** Dong Li, Ting Liu, Xiaobing Zuo, Tao Li, Xiangyun Qiu, Alex Evilevitch

**Affiliations:** 1 Physics Department, Carnegie Mellon University, Pittsburgh, PA 15213, USA; 2 X-ray Science Division, Advanced Photon Source, Argonne National Laboratory, Argonne, IL 60439, USA; 3 Department of Physics, The George Washington University, Washington, DC 20052, USA; 4 Department of Biochemistry and Structural Biology, Lund University, SE-221 00 Lund, Sweden

## Abstract

We have recently found that DNA packaged in phage *λ* undergoes a disordering transition triggered by temperature, which results in increased genome mobility. This solid-to-fluid like DNA transition markedly increases the number of infectious *λ* particles facilitating infection. However, the structural transition strongly depends on temperature and ionic conditions in the surrounding medium. Using titration microcalorimetry combined with solution X-ray scattering, we mapped both energetic and structural changes associated with transition of the encapsidated *λ*-DNA. Packaged DNA needs to reach a critical stress level in order for transition to occur. We varied the stress on DNA in the capsid by changing the temperature, packaged DNA length and ionic conditions. We found striking evidence that the intracapsid DNA transition is ‘switched on’ at the ionic conditions mimicking those *in* *vivo* and also at the physiologic temperature of infection at 37°C. This ion regulated on-off switch of packaged DNA mobility in turn affects viral replication. These results suggest a remarkable adaptation of phage *λ* to the environment of its host bacteria in the human gut. The metastable DNA state in the capsid provides a new paradigm for the *physical evolution* of viruses.

## INTRODUCTION

The idea that genes could be switched on and off has revolutionized molecular biology (1,2). It originates from studies on bacteriophage λ, which allowed establishment of the temperature induced mechanism of lysogeny-lysis transition, called ‘A Genetic Switch’ (1). In this work, we discovered for the first time, that the temperature and ionic composition in the surrounding medium of bacterial cells not only switch phage λ genes on and off (1,3–4), but also serve as an on-off switch for DNA structural transition in λ-capsid. (Previous studies have shown a salt-dependent behavior of repressor DNA-binding affinity in temperate bacteriophages (3,4), indicating that lysis-lysogeny switches in phage λ are both temperature- and salt-sensitive). This transition increases DNA mobility, which facilitates the initiation of genome ejection from phage into a cell and thus leads to infection (5,6).

*Virion metastability* is one of the central concepts in virology (7). It implies that the virus, in order to successfully replicate, must be sufficiently *stable* to prevent spontaneous release of its genome outside the cell between infection events, and at the same time be *unstable* enough to release its genome during infection. Viral particles are therefore not inert structures and have not attained the minimum free energy conformation, separated by an energetic or kinetic barrier, prior to cell attachment and entry (7). Thus, viral structure plays an active role in genome delivery to the host cell. Viral metastability is mostly associated with structural transformations in the nucleocapsid and/or surrounding lipid envelop in response to changes in the virion's environment (7). However, this does not apply to motor-packaged double-stranded (ds) DNA viruses (e.g. dsDNA phages and Herpesviruses) whose capsid remains intact after the genome is released into a cell through a portal opening in the capsid structure (8–11). We recently found that it is the encapsidated DNA rather than the capsid itself in these viruses that is metastable (5,6). Specifically, we have shown that dsDNA packaged in phage λ (5) and human Herpes Simplex virus 1 (HSV-1) (6) undergoes a solid-to-fluid like structural transition facilitating initiation of viral genome ejection from the capsid during infection.

dsDNA packaged in both HSV-1 and phage λ is two orders of magnitude longer than the diameter of the capsid. This tight genome confinement leads to strong DNA–DNA repulsions and bending stress on the genome generating pressure of tens of atmospheres on the capsid walls (9,10). The encapsidated DNA is at the extreme end of the packing limit (reaching packing fraction of 55% by volume) (12–15), with only ∼7–12 Å of interhelical surface separation (15). DNA at this packing density assumes a hexagonally ordered structure with very restricted mobility (5–6,16–17). This is caused by interhelical electrostatic sliding friction (Coulomb friction) (18–20), occurring from dragging closely packed negatively charged DNA helices past other helices.

**Figure 1.**
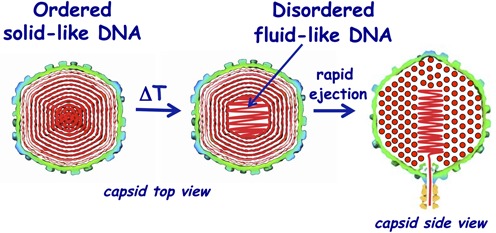
DNA disordering transition is suggested to take place close to the center of the capsid, where DNA is more stressed due to the stronger bending and larger packing defects (5). The DNA transition occurs when the temperature is increased. As a result, the increased mobility of the encapsidated viral DNA provides faster genome release in the cell, facilitating the infection process. Two schematic images on the left show the cross-sections of the top view of the capsid. DNA closer to the center of the capsid is likely to be ejected first since it is the last DNA portion to be packaged in the capsid during the phage assembly, due to the dsDNA bending stress constraints. This is illustrated with the side view cross-section of the capsid (right image). The schematic illustration of DNA inside the capsid shows the ordering of an averaged DNA structure and not the arrangement of individual DNA strands.

We have demonstrated for phage λ (5) that DNA layers closest to the capsid's center undergo a disordering transition induced by an increase in temperature. This occurs from an increase in interstrand repulsions, due to DNA packing defects (5), leading to stronger genome stress (as described in details below). DNA disordering at the transition temperature leads to a locally lower packing density in the center of the capsid, maximizing DNA–DNA spacings which in turn reduces interstrand repulsions. This leads to a more mobile, or fluid, DNA state which can be readily ejected from the capsid (5,6). This observation is schematically illustrated in Figure 1. Thus, below the transition temperature, viral DNA is trapped in a solid-like metastable state inside the capsid, which delays the initiation of its spontaneous release from the capsid and deactivation of the virion. An increase in temperature induces the necessary mobility of the packaged genome, facilitating its release and infection of the bacterial cell. Indeed, using the single-molecule fluorescence measurements below and a plaque assay, we confirmed a marked increase in the rate of infection spread at temperatures above that of DNA transition (5). However, it is important to emphasize that we also found that the transition temperature is directly coupled to the critical DNA stress value in the capsid (5,6). The encapsidated genome stress is, in turn, regulated by DNA counterions and packaged DNA density, both affecting the repulsive interactions between DNA helices (21,22). This raises a fundamentally important question, whether the packaged DNA density in phage λ and the ionic conditions *in* *vivo* lead to an intracapsid DNA transition occurring at the physiological temperature of infection, i.e. 37°C corresponding to human body temperature (λ replicates in *Escherichia coli* in the human gut) (23,24)? Only then can the observed DNA transition phenomenon in phage have physiological relevance and impact on the efficiency of viral replication.

With this aim, we investigate DNA transition in phage λ as a function of packaged DNA density and at varying ionic conditions mimicking those *in* *vivo*. We mapped both energy (using Isothermal Titration Calorimetry, ITC) and structure (using solution Small Angle X-ray Scattering, SAXS) of the packaged λ-genome and found a remarkable paradigm of physical adaptation of viruses to their host. Our data show that disordering of the packaged DNA in λ-capsid (which is linked to increased intracapsid DNA mobility (5,6)) is ‘*switched on*’ for its efficient release from the capsid at the most favorable ionic and temperature (37°C) conditions for *E. coli* infection. Furthermore, the transition occurs only at the wild-type (WT) λ-DNA length (48500 bp) density packaged in the capsid, as opposed to the shorter DNA length phage λ mutants. This suggests that this ion-regulated DNA disordering transition mechanism is likely to be a part of the phage λ replication decision.

## MATERIALS AND METHODS

### Phage λ and LamB purification

WT bacteriophage λ cI857, with a genome length of 48.5 kb was produced by thermal induction of lysogenic *E. coli* strain AE1 derived from the S2773 strain. Two phage λ mutants with shorter genome lengths (78 and 94% of the WT DNA length) were produced using a similar procedure. The receptor was the LamB protein purified from pop 154, a strain of *E. coli* K12 in which the *LamB* gene has been transduced from *Shigella sonnei* 3070. Phage and LamB purification details are described in Supplementary Materials (10,25).

### Isothermal titration calorimetry (ITC)

All calorimetric measurements were performed using the MicroCal iTC200 system manufactured by GE Healthcare, Life Sciences. The details of phage DNA ejection enthalpy measurements are described in Supplementary Materials (5,26).

### Analysis of SAXS measured DNA diffraction peak position and area

Small angle X-ray scattering (SAXS) measurements were carried out at the 12-ID B station at the Advanced Photon Source at Argonne National Laboratory. A 12-KeV X-ray beam was used to illuminate the sample with an overall scattering vector *q* range from 0.006 to 0.850 Å^−1^. A total of 120 μl of phage solution (∼5 × 10^13^ pfu/ml) was injected into a flow-through glass capillary and the solution was oscillated during the SAXS measurement with a flow rate of 10 μl/s. Forty scans with 1 s X-ray exposure time were collected and averaged for each sample. A buffer solution of the dialysis buffer for phage samples was measured using the same SAXS setup, which was further subtracted as the background. After the background subtraction, the scattered intensity *I* versus *q* was plotted and the DNA peak region was truncated from 0.18 Å^−1^ to 0.33 Å^−1^. This DNA diffraction peak was fitted with a Gaussian curve plus a linear background using function (1), where *q*_0_ is the peak center, *w* is the peak width, *A*_0_ is the peak area, *k* is the slope of the linear background and *c* is the offset.

(1) \begin{equation*} I = \left( {\frac{{A_0 }}{{w \times \sqrt {\pi /2} }}} \right)e^{ - 2\left( {\frac{{q - q_0 }}{w}} \right)^2 } + kq + c \end{equation*}

The DNA peak area *A*_0_ was chosen as the most convenient measure of the ordered DNA strands, because it includes a temperature factor or the displacement parameter, which signifies the drop in the diffraction peak intensity due to the thermally induced vibration or displacement of the scattering centers. Further details are provided in Supplementary Materials (5).

### Single-molecule fluorescence measurements of phage ejection ensemble kinetics

Phage particles were imaged using a Nikon 2000E2 microscope with a spinning disk confocal scan head (Yokagawa Industries, Tokyo, Japan). See details in Supplementary Materials.

## RESULTS AND DISCUSSION

### Effect of packaged genome density on intracapsid DNA transition

Temperature is a parameter that is rarely varied in biophysical measurements on viruses. Yet, temperature is directly pertinent to the ability of viruses to survive outside of their hosts and multiply within them. As mentioned above, we found that WT DNA length packaged in phage λ undergoes a disordering transition in response to a temperature increase (5). This structural change leads to a more mobile DNA state inside the capsid, which facilitates the initiation of its rapid release into a cell during infection (5,6). In this section we investigate how the packaged genome length in λ affects the structural transition behavior. Using ITC and SAXS we are analyzing changes in the energy and structure of the encapsidated DNA with 78, 94 and 100% of the WT λ-DNA length between 10 and 40°C. The ionic conditions in these measurements are set by 10 mM MgCl_2_ Tris-buffer (50 mM Tris was used in all the measurements below). As discussed in Section 2 below, this Mg-concentration mimics the optimum Mg-concentration in the culture medium required for *E. coli* growth (by preventing ribosome degradation) (27) and it also provides the most efficient phage λ adsorption to bacterial cells, maximizing the subsequent infection (28,29). [Intracellular Mg-concentration is actively controlled in *E. coli* (27,30–31) and is estimated to also stay within 5 mM (32) to 10 mM (33) of free Mg^2+^].

**Figure 2.**
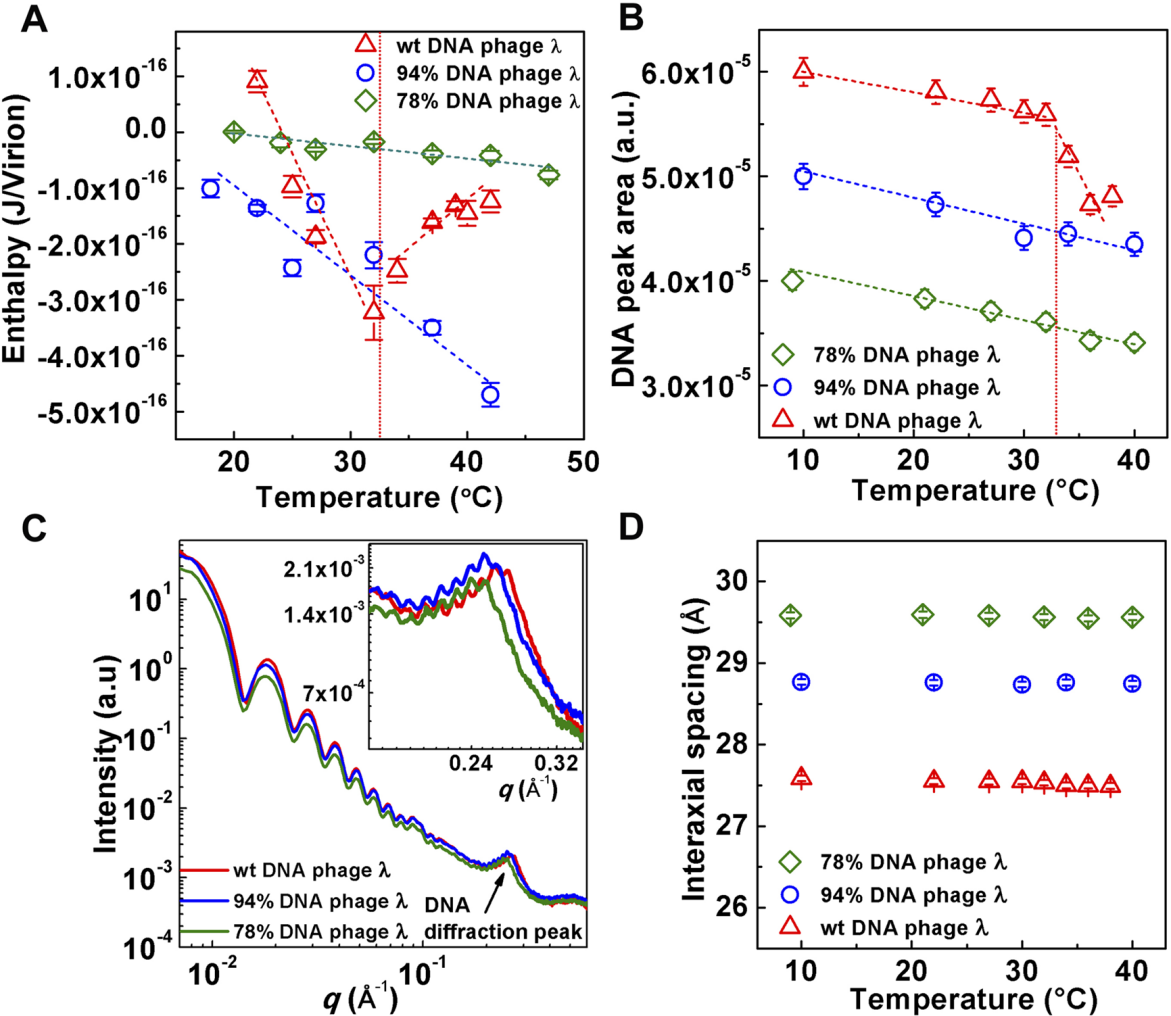
ITC and SAXS data collected in 10 mM MgCl_2_ Tris-buffer. (**A**) Enthalpy of DNA ejection per virion (J) versus temperature for phage *λ* with 100, 94 and 78% of WT packaged *λ*-DNA length. Dashed lines are drawn to guide the eye. *ΔH_ej_* values were obtained as an average of five to six independent measurements for each sample. Vertical error bars are SEs. (**B**) DNA–DNA diffraction peak area as a function of temperature for 100, 94 and 78% of WT DNA length packaged in phage *λ*. The peak area is obtained by fitting the scattering curve with a Gaussian function with linear background subtraction. The vertical error bars are from the non-linear fitting. A constant has been added to each set of area values corresponding to one DNA length phage mutant in order to vertically separate the data for visual comparison. The dashed line is drawn to guide the eye. (**C**) Radially averaged scattered intensity versus the scattering vector *q* for 100, 94 and 78% of WT DNA length phage *λ* mutants at 22°C. Inset figure shows the zoom-in of the DNA diffraction peak for three DNA length λ-phages. The scattering profile at lower *q* is attributed to the protein capsid. (**D**) DNA–DNA interaxial spacing *d* as a function of temperature for 100, 94 and 78% of WT *λ*-DNA length packaged in phage *λ*. Vertical error bars are from the non-linear fitting of the DNA diffraction peak with a Gaussian function with linear background subtraction.

We designed a new micro-calorimetric assay that allows direct measurements of the internal energy of the confined viral genome (5). Using micro-ITC, the enthalpy change *(ΔH*) associated with DNA ejection from phage λ is measured as heat released when concentrated phage particles are titrated into a LamB receptor solution, which triggers DNA ejection *in* *vitro*. Since the total volume of the system does not change during the DNA ejection and the pressure is constant, the internal energy and the enthalpy are approximately equal (5,26). The temperature in the reference cell is continuously equilibrated to that of the sample cell after each titration of phage in LamB solution. The differential power between the reference cell and the sample cell is recorded in microcalories per second (see Supplementary Figure S1 in Supplementary Materials). Integration of the area under the heat change peak over time provides the reaction enthalpy, which includes DNA ejection from the phage, mixing of phage in LamB solution, dilution of LamB and the pressure–volume work associated with titration of one volume into another. (Enthalpy contributions from binding of LamB to the phage tail and any subsequent protein conformational changes leading to initiation of DNA ejection are several orders of magnitude lower than the enthalpies listed above (34)). These other contributions to the enthalpy change that are not arising from the enthalpy of DNA ejection *(ΔH_ej_*) are measured separately by titrating phage into the buffer, buffer into LamB solution and buffer into buffer. These values are subtracted from the total enthalpy change *(ΔH*). The enthalpy change associated with DNA ejection from phage is *ΔH_ej_(T) = ΔH(T)*_DNA ejected_ *– ΔH(T)*_DNA inside phage_*. ΔH_ej_(T)* was measured in the temperature range between 18 and 42°C. Figure 2A reveals a discontinuity in the approximately linear dependence of *ΔH_ej_* on temperature occurring at T* ∼33°C for WT DNA length ejection from phage λ in 10 mM MgCl_2_ Tris-buffer. The discontinuity demonstrates an abrupt transition that can be attributed to the DNA inside the capsid. [Differential scanning calorimetry analysis of free λ-DNA in solution confirmed that there is no structural transition in this temperature range, with double-to-single stranded DNA melting occurring at significantly higher temperatures (35,36)]. Prior to the transition, the absolute value of the ejection enthalpy change $|\Delta H_{ej} (T)|$ shows a strong linear increase with increasing temperature. $|\Delta H_{ej} (T)|$ increases nearly four times when the temperature is raised from 22 to 32°C. This increase in the internal energy indicates an increase in the stress of the confined genome as temperature is being raised. At the transition temperature, T*, the internal energy is reduced by almost half, suggesting partial relief of the stressed state. After the transition, $|\Delta H_{ej} (T)|$ shows only weak temperature dependence when the temperature is further increased to 42°C, see Figure 2A. This observation demonstrates that the DNA inside λ capsid can exist in two energy states.

Our data show that the critical genome stress is reached at temperature T* and is required for the structural transition to occur. This suggests that varying the initial DNA stress inside the capsid should determine whether the transition will occur within the studied temperature range. We test this hypothesis by repeating the ITC measurements above for shorter packaged genome length λ-mutants, 78 and 94% of the WT λ-DNA length, while keeping the buffer conditions the same. DNA pressure inside λ capsids is significantly reduced when shorter DNA length is packaged (15 atm in 78% λ-DNA phage versus 35 atm in WT λ-DNA phage) (21). Lower DNA density in the capsid has larger spacings between packaged DNA helices, resulting in weaker repulsive interactions and lower genome stress (21). Indeed, we found that no DNA transition occurred inside the capsid for these λ-mutants with shorter packaged DNA length than WT λ-DNA (within the investigated temperature range), see Figure 2A. This demonstrates that there is a strong dependence between the internal genome stress and the structural DNA transition in phage λ. Next, we investigate how the structural changes of DNA in the capsid are associated with the observed energy transition using SAXS.

Solution SAXS provides direct structural information about the encapsidated genome (5,12,37). Capsid proteins and packaged DNA have well-resolved scattering profiles (5,6). Figure 2C shows integrated scattering intensity, *I*, versus scattering vector *q* for all three packaged genome length λ particles (78, 94 and 100% of WT DNA length). In the lower *q* region (0.007Å^−1^ to 0.1Å^−1^), the scattering profile originates from the highly symmetrical icosahedral phage capsids. The single peak with the small oscillating ripples on its top at higher *q* (between 0.2 Å^−1^ to 0.3 Å^−1^) is due to the diffraction from the encapsidated ordered DNA strands. The short-range DNA–DNA interaxial spacings determine the position of the DNA diffraction peak, while the area of this peak indicates how well the DNA strands are aligned relative each other, i.e. it provides information on the total number of ordered DNA base pairs of the encapsidated genome (5,12). When DNA inside the capsid becomes less ordered, the DNA peak area decreases as a result of less coherent diffraction. If the genome is completely disordered, the DNA diffraction peak disappears. The average DNA–DNA interaxial spacing, *d*, is calculated using $d = \frac{{4\pi }}{{\sqrt 3 q}}$, assuming the hexagonal packing structure of DNA (12). The position of the DNA diffraction peak gradually shifts to lower *q* values as the packaged DNA length decreases from 100 to 78% of WT λ-DNA length (see the inset in Figure 2C), indicating that the interaxial distance between packaged DNA strands increases, shown in Figure 2D. The DNA interaxial distance was nearly constant between 10 and 40°C for each DNA length λ-mutant, Figure 2D.

The encapsidated DNA structure and interaxial distance between ordered DNA strands is determined by DNA–DNA electrostatic and hydration repulsive interactions (38–40), the genome bending stress, as well as packing defects (15,37,41). Intracapsid confinement requires DNA to bend along radii that are energetically unfavorable given the stiffness of dsDNA (42,43). To relieve the bending stress, helices are packed closer to the capsid wall, increasing the bending radius and also decreasing the DNA–DNA spacing. At the same time, the repulsive DNA–DNA interactions attempt to push DNA strands as far from each other as possible, maximizing the interstrand separations (15) and filling the entire capsid volume. With shorter λ-DNA length packaged in the capsid, the DNA–DNA distance is increased in order to minimize the interstrand repulsions. As we have previously found, the entire capsid volume is filled with DNA extending all the way to the center of the capsid (15). Supplementary Figure S2 in Supplementary Materials shows cryo electron microscopy (cryo-EM) single particle reconstructions of λ-capsids filled with 78 and 100% of WT DNA length. Starting from the capsid walls, there are well ordered, multiple concentric DNA layers. The layers are evenly spaced indicating that DNA has adapted an ordered repetitive structure characteristic of a liquid crystalline state. However, toward the center of the capsid, the ordered layers disappear suggesting a less ordered DNA structure than in the periphery of the capsid (9).

We have previously shown that with increasing temperature, DNA–DNA repulsive interactions are not significantly affected (see Supplementary Figure S3 in Supplementary Materials) (5). However, an increase in temperature will decrease the DNA persistence length (44), leading to less bending stress. (Persistence length defines the stiffness of a polymer, describing the minimum radius of curvature it can adopt by the available thermal energy. Bending it to a smaller radius requires additional work). If the bending stress decreases due to repulsive interactions, the DNA will expand towards the center of the capsid, increasing interstrand separations. However, Figure 2D shows that the spacing remains essentially constant for all three λ-DNA length mutants in 10 mM MgCl_2_ Tris-buffer, suggesting that the temperature induced decrease in the bending stress is insufficient to affect the balance between the repulsive forces and the bending stress on the encapsidated genome. (The effect of increased interaxial spacing with increasing temperature due to a decrease in the bending stress will be demonstrated below under different ionic conditions than those above). Besides repulsions and bending stress, when DNA is bending inside the capsid, the initial correlation between two helices that have slightly different radii of curvature is lost, and the mutual orientation between helices must be re-established. This leads to the packing defects that are absent for linear packaging of DNA in solution. Packing defects are required in order to re-establish a favorable phosphate–phosphate ‘phasing’ of helices, reducing the repulsive interactions due to bending (15,41,45). Indeed, ITC data in Figure 2A show a progressive increase in the packaged DNA energy with increasing temperature prior to DNA transition. To relieve this increasing DNA stress in the capsid, the genome undergoes a disordering transition when the maximum stress value is reached at temperature T*. Figure 2B shows the area of DNA scattering peak versus temperature, reflecting the overall ordering of the packaged genome for all three DNA length λ-mutants. For WT λ-DNA length phage at T*∼33°C, the DNA diffraction peak area undergoes a sudden drop. This area drop signifies a loss of the amount of ordered DNA inside the capsid and occurs precisely at the transition temperature observed with ITC in Figure 2A. Our previous data suggest that a disordering transition takes place closer to the center of the capsid, where packing defects are larger than for DNA closer to the capsid wall (5), as illustrated in Figure 1. With increasing temperature the DNA bending stress becomes smaller. This allows a fraction of the ordered DNA layers closest to the capsid's center (where DNA bending stress is stronger) to undergo a disordering transition. The disordered DNA in the capsid center will have a locally lower packing density, which maximizes DNA–DNA spacings and simultaneously reduces the repulsive interactions. This yields an overall lower energy state of the encapsidated genome and increases its mobility, which in turn facilitates DNA release during infection (5).

At the same time, in agreement with ITC results, SAXS data in Figure 2B show that shorter λ-DNA length mutants (78 and 94% of WT DNA length phages), do not undergo a disordering transition within the investigated temperature range of 10–40°C. The DNA peak area shows only a weak linear decrease with increasing temperature for these shorter DNA λ-mutants without an abrupt drop. This result confirms that in order for DNA transition to occur, the encapsidated genome has to reach the critical stress value first, which is not achieved if the DNA packing density is lower than in the WT phage λ. It is remarkable that intracapsid genome transition occurs within the physiologic temperature range only when packaged DNA in the λ-capsid has a length corresponding to that of the WT λ-genome. While shorter genome length λ-mutants will also experience reduced electrostatic sliding friction and therefore result in a more mobile DNA state in the capsid, those λ-mutants will have reduced capsid pressure (21). We have shown that the reduced DNA pressure in these phage λ DNA mutants leads to a markedly reduced probability of infection due to the lack of driving force for DNA ejection (e.g. infectivity is reduced by ∼30% for 78% DNA length λ compared to the WT DNA phage λ) (46). The specific WT DNA length (48.5 kbp) in phage λ appears to provide the unique genome structure with a stress value capable of both driving DNA release from the capsid and creating a metastable DNA state required for solid-to-fluid like DNA transition to occur. These physical parameters are required for efficient DNA release from phage λ during infection.

In addition, it should be noted that sharp bending or kinking of dsDNA occurring in the center of the capsid can excite local melting of a few base pairs, unwinding the DNA where the sharp bending occurs (47–50). It has been shown that at a critical radius of DNA curvature of 3.5–4.0 nm, the bending stress disrupts the helical DNA structure, with dsDNA opening up and being digested by a single-strand-specific endonuclease (50). Excitation of melted base pairs provides flexible local joints of DNA, which relax both bending and twisting stress of DNA, reducing the order of packing. This in turn is consistent with the more fluid-like behavior of the packed DNA observed after the transition. Low apparent DNA bending stiffness for sharply bent DNA has been previously reported (48,49). Excitations of flexible DNA defects were also theoretically analyzed (51,52). *ΔH* for DNA transition derived from our ITC measurements for WT phage λ has a value of ∼1.8 × 10^4^ kT/virion at 33°C in 10 mM MgCl_2_ Tris-buffer. Since DNA transition does not occur in the 94% of WT λ-DNA length phage λ mutant but does occur in the WT λ-DNA length phage λ, it is reasonable to assume that the transition occurs within the 6% of the full λ-DNA length (48 000 bp) packaged in λ-capsid. This gives a lower bound estimate for the DNA transition enthalpy of ∼6 kT/bp. This value is significantly higher than both DNA bending energy at small radii (e.g. a radius of curvature of 2.5 nm results in bending energy of ∼1.3 kT/bp) (53) and DNA kinking energy of ∼0.8 kT/bp in the presence of Mg^2+^(54). This suggests that local DNA melting induced by a temperature increase resulting in an observed disordering transition in the center of the capsid is possible. However, earlier Raman spectroscopy studies of DNA packaged in phage P22 with DNA packing density similar to that in λ, have not detected any base unpairing or strand separation in the packaged DNA structure at 25°C under buffer conditions similar to ours (55). At the same time, the P22 portal structure significantly protrudes into the capsid interior (56), unlike in phage λ, which can interfere with the packaged DNA structure.

Thus, we have shown that intracapsid DNA transition is strongly correlated with the internal DNA stress. However, the DNA stress in the capsid is regulated by both packaged DNA length and by the concentration and nature of the DNA counterions freely diffusing through the capsid wall of most viruses (22). This fact leads us to the next important question which is whether extra- and intra-cellular ionic conditions in the bacterial host are favorable for DNA to undergo a transition in the phage λ capsid close to the physiologic temperature of infection? The effect of ionic conditions on the DNA transition temperature is investigated in the next section.

### Effect of ionic conditions on DNA transition temperature

While variation in the monovalent ion concentration has a small influence on intra-capsid DNA stress (22), polyvalent cations present in the bacterial cytoplasm, such as polyamines and Mg^2+^, have been shown to have a strong effect on the repulsive interactions between packaged DNA helices (22,37). Since the free polyamine concentration in cells is very low as most polyamines are bound to cellular DNA and RNA (57–59), we are specifically interested in the effect of the Mg-ion concentration on the DNA transition temperature in phage λ at concentrations similar to those of extra- and intracellular Mg^2+^ *in* *vivo* (27–28,30–33).

Mg-ions are essential for both cellular metabolism (e.g. enzyme activity, protein synthesis, preservation of ribosome and nucleic acid structures) (33,60–62), as well as the phage λ infectious cycle (28,29). Intracellular Mg-concentration is actively controlled in *E. coli* (30), where Mg^2+^ is mainly supplied by the CorA protein transporter (31). Lack of Mg^2+^ in *E. coli* growth medium induces ribosome degradation, with ribosomes becoming inactivated at extracellular Mg-concentrations below 10 mM (27). Therefore, normal growth medium for *E. coli* has a Mg-concentration of 10 mM. Furthermore, it has been shown that a concentration of 10–20 mM of Mg^2+^ ions in the extracellular solution is critically important for optimum adsorption and infection of *E. coli* by phage λ (28). Mg-concentrations below or above 10–20 mM had a strong negative effect on the number of bacterial cells that could be infected. A substantial fraction of the total intracellular Mg^2+^ in *E. coli* is bound to cellular proteins and nucleic acids. The total Mg^2+^ concentration (bound and free) in *E. coli* cytoplasm varies slightly depending on different growth phases (∼50 mM in the exponential phase and ∼40 mM in the stationary phase) (33). The free intracellular Mg-concentration is estimated to be around 5 (32) to 10 mM (33,63–64). Interestingly, a similar Mg-concentration (∼10 mM Mg^2+^) has also been shown to provide optimum conditions for DNA packaging in phage λ (29,65).

As mentioned above, since the Mg-concentration affects DNA stress in the capsid, it also strongly influences the transition temperature of the encapsidated genome (5). Therefore, we investigate whether the physiologic Mg-concentrations described above provide optimum conditions for phage λ infectivity *in* *vivo* by facilitating DNA transition in the λ capsid close to the temperature of infection (37°C). Such a unique correlation between the Mg-concentration and DNA transition temperature would suggest that the intracapsid DNA transition is an important regulatory mechanism for viral replication.

**Figure 3.**
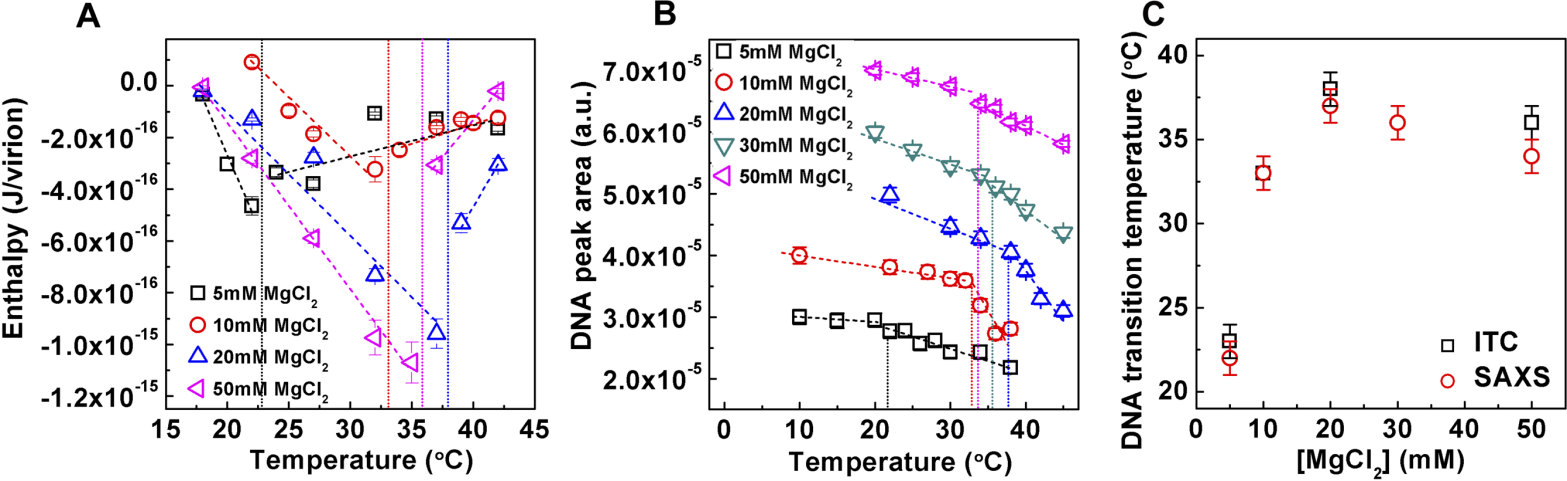
(**A**) Enthalpy of DNA ejection per virion (*J*) from WT DNA phage *λ* versus temperature, Δ*H*_ej_, in MgCl_2_ Tris-buffers with varying MgCl_2_ concentrations. Dashed lines are drawn to guide the eye. The vertical dashed lines indicate the DNA transition point. Vertical error bars are SEs. (**B**) DNA diffraction peak area as a function of temperature for WT DNA phage **λ** in MgCl_2_ Tris-buffers with varying MgCl_2_ concentrations. A constant has been added to each set of area values corresponding to one Mg-concentration in order to vertically separate the data for visual comparison. The vertical dashed lines indicate the DNA transition point. The vertical error bars are from the non-linear fitting. (**C**) Comparison between DNA transition temperatures determined by ITC and SAXS as a function of MgCl_2_ concentration in Tris-buffer.

We are using ITC and SAXS assays described above to determine DNA transition temperatures for WT λ-DNA length phage in MgCl_2_ Tris-buffer with MgCl_2_ concentrations varied between 5 and 50 mM, see Figure 3. The intracapsid DNA transition occurs at temperature T*, corresponding to either a discontinuity in the linear dependence of *ΔH_ej_* on temperature (Figure 3A) or to an abrupt change in the linear decay of the DNA diffraction peak area versus temperature measured by SAXS (Figure 3B). DNA transition temperatures as a function of Mg-concentration determined by both techniques are in good agreement with each other and are summarized in Figure 3C. Increasing the Mg-concentration will initially significantly reduce the strength of the interstrand repulsive interactions in the capsid due to the counterion screening of the negative charges between packaged DNA helices (15,22). However, we have previously shown that at DNA packing density in phage λ, the screening effect of Mg-ions will become progressively smaller due to the counter-ion saturation (22). Furthermore, if the Mg-ion concentration continues to increase, the counterions are forced into regions on the helices that begin to increase the effective repulsions between packaged DNA strands. Thus, there is a minimum in interstrand repulsive interaction versus Mg-concentration (22,66).

Figure 3C shows that the DNA transition temperature T* is at first significantly increased with increasing Mg-concentration, from T*∼22°C at 5 mM MgCl_2_ Tris-buffer to T*∼37°C at 20 mM MgCl_2_ Tris-buffer. However, increasing the Mg-concentration further has an opposite effect on the DNA transition temperature, showing a weak decrease in T* between 20 and 50 mM MgCl_2_. T* reaches ∼35°C at 50 mM MgCl_2_, see Figure 3C. This variation in the transition temperature correlates well with previously observed non-monotonic variation in the DNA stress in the capsid with increasing Mg-concentration (22), as described above. Initially, as the Mg-concentration is increased between 5 and 20 mM, the internal DNA stress is reduced leading to a higher transition temperature. That is, a higher temperature is required to reach the critical DNA stress limit in order to overcome the energetic barrier triggering the structural genome transition. Once the Mg^2+^-ion saturation yielding maximum counterion screening between the packaged DNA helices occurs at ∼20 mM (22), the interstrand repulsive interactions start to increase again with increasing Mg-concentration. This results in a decrease of T*, since the critical intracapsid DNA stress level required for transition is now achieved at a lower temperature. Thus, while T* varies significantly with Mg^2+^ concentration, the most favorable Mg-concentration for *E. coli* growth (27) and for phage λ infection of *E. coli* (∼10–20 mM) (28) triggers DNA transition in the capsid precisely at the physiologic temperature of infection (∼37°C). This striking correlation confirms our assumption, namely, that the intracapsid DNA transition mechanism in λ is evolutionarily adapted to both the ionic environment and temperature of its host, suggesting its significance for viral replication.

In the next section we investigate how polyvalent ions in the host solution can switch the DNA transition in phage λ on and off by directly affecting the interstrand repulsions, and consequently, the stress level of the encapsidated genome. Since DNA transition markedly affects the ability of the virion to eject its DNA into a cell (5), these results demonstrate how variations in the extracellular ionic environment can lead to favorable as well as inhibiting conditions for infectivity.

### Switching intracapsid DNA transition on and off

As discussed above, intracapsid DNA transition is triggered by a temperature increase. However, DNA in the capsid has to reach the critical stress value at T* before the transition can occur. The overall DNA stress can in turn be varied by packaged DNA length (21) or by polyvalent cations (22) diffusing into the capsid and affecting the interstrand repulsive interactions. We observed that by varying either of these two parameters leads to variation in the internal DNA stress and transition temperature. This suggests that the intracapsid DNA transition can be switched on and off for the same viral particle within the physiologically relevant temperature range studied here (10–45°C). To demonstrate this assumption, we selected 78 and 100% WT DNA length phage λ in 10 mM MgCl_2_ Tris-buffer as our reference systems. The 78% WT DNA length λ does not display an intracapsid DNA transition (Figure 2A and B), while 100% WT DNA length phage λ undergoes a DNA transition at ∼33°C (Figure 2A and B).

**Figure 4.**
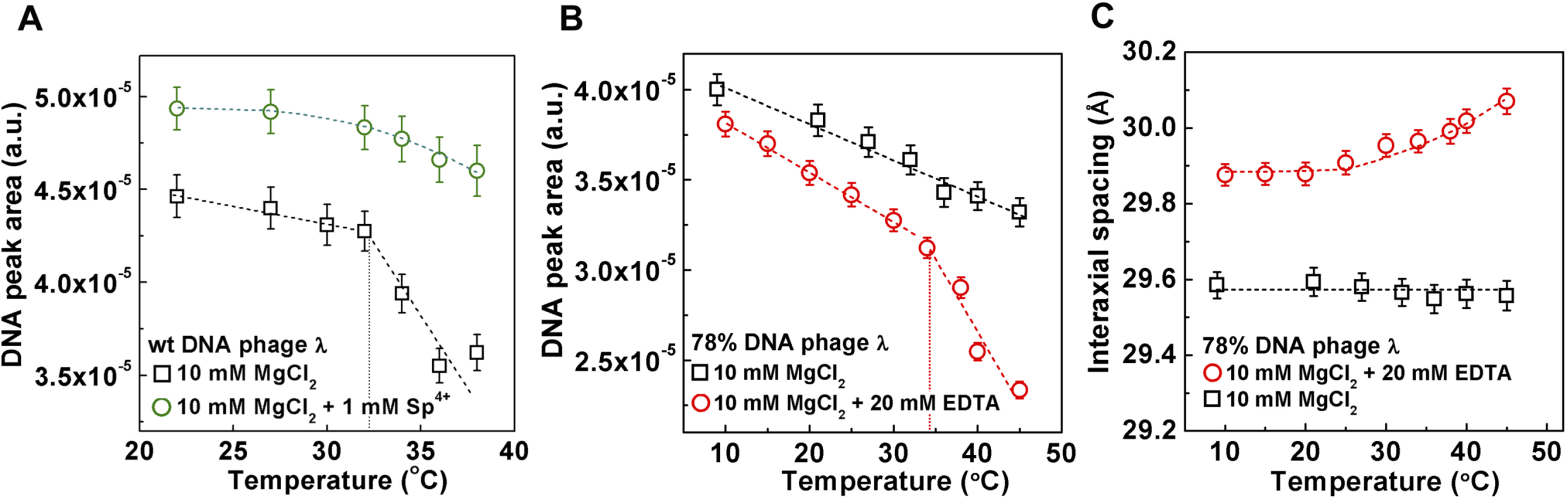
(**A**) DNA diffraction peak area as a function of temperature for WT DNA phage λ in 10 mM MgCl_2_ Tris-buffer without and with 1 mM spermine (4+). (**B**) DNA diffraction peak area as a function of temperature for 78% of WT DNA length phage λ in 10 mM MgCl_2_–Tris buffer without and with 20 mM EDTA. (**C**) DNA–DNA interaxial spacing *d* as a function of temperature for 78% of WT DNA length phage *λ* in 10 mM MgCl_2_ Tris-buffer without and with 20 mM EDTA. In A, B and C, the vertical error bars are from the non-linear fitting of the DNA diffraction peak with a Gaussian function with linear background subtraction. The dashed line is drawn to guide the eye.

In the first set of measurements, we added 1 mM of spermine (4+) ions to the WT DNA length phage λ to introduce attractive interactions between packaged DNA strands (15,22,38). This allows re-establishing of inter-strand packing defects (that were hindered by the increased temperature) and reduces the overall DNA stress. The DNA diffraction peak area now shows only slight variation in the entire temperature range (20–40°C), and no abrupt DNA transition is observed, in contrast to WT DNA phage λ without added sperimine (4+), see Figure 4A. Thus, the DNA transition in the capsid has been switched off with spermine (4+) ions, where the increasing DNA repulsive interactions with increasing temperature (resulting from hindering of the packing defects) are now offset by the spermine (4+) induced attractive interactions. The DNA stress in the capsid is therefore insufficient for DNA transition to occur.

Next, we investigate if DNA transition can instead be switched on in the 78% WT DNA length phage λ. As shown above, 78% WT DNA length phage λ in 10 mM MgCl_2_ Tris-buffer does not display a DNA transition between 10 and 40°C due to insufficient DNA stress in the capsid, see Figure 2A and B. Through addition of excess ethylenediaminetetraacetic acid (EDTA), we chelate Mg-ions (20 mM EDTA added to 10 mM MgCl_2_ Tris-buffer solution), which increases repulsion between packaged DNA strands and leads to a higher DNA stress in the capsid. This increase in DNA stress in the 78% WT DNA length phage λ appears to be sufficient to trigger DNA transition at T* ∼36°C. Figure 4B shows the DNA diffraction peak area versus temperature for 78% WT DNA length phage λ with and without EDTA. There is an abrupt drop in the DNA peak area when EDTA is added, demonstrating a DNA disordering transition. We conclude that DNA transition in a viral capsid can be switched on and off by varying the polyvalent counterion concentration, which affects the repulsive interactions between packaged genome strands.

Figure 4C shows variation in the interaxial DNA–DNA distance in the capsid as a function of temperature for 78% WT DNA length λ in 10 mM MgCl_2_ Tris-buffer with and without EDTA addition. As expected the interaxial distance is increased when EDTA is added. This is caused by increased repulsive interactions between packaged DNA strands when Mg-ions are chelated by EDTA. However, it is interesting to note that with EDTA added, the DNA interaxial distance is increasing with temperature, while it remains essentially constant with increasing temperature without EDTA addition, see Figure 4C. As described above, the balance between the bending stress and the interstrand interactions determines the interaxial distance between the ordered DNA strands (15,37). With temperature increasing from 10 to 45°C, interstrand interactions remain essentially unchanged (see Supplementary Figure S3), but DNA persistence length is decreased by almost 30% (44), making DNA more flexible, which leads to smaller bending stress. Reduced bending stress should in turn cause DNA–DNA spacings to increase due to repulsive interactions striving to push the DNA closer to the center of the capsid (where the bending stress is higher than in the periphery due to the higher curvature). However, if the temperature induced change in the bending stress is insufficient to affect the force balance between interaction and bending forces, DNA spacing will remain unchanged. This is indeed the case for 78% WT DNA length phage λ without EDTA added in 10 mM MgCl_2_ Tris-buffer. The DNA interaxial distance remains at ∼29.6 Å between 10 and 45°C, see Figures 2D and 4C. Interestingly, when EDTA is added to the same phage solution, the stronger DNA–DNA repulsive forces (due to chelated Mg^2+^) start to dominate over the opposing forces on the DNA due to the bending stress, resulting in an increase in DNA interaxial spacing with increasing temperature. At first there is almost no change in DNA spacing between 10 and 20°C, but later, as bending stress becomes smaller, we observe a progressive increase in interaxial spacing from 29.6 Å at 20°C to 30.1 Å at 45°C, see Figure 4C. These data illustrate a delicate interplay between bending and interaction forces on the packaged genome, which determine its structure and the overall stress level.

In the last section below, using single-molecule fluorescence measurements, we demonstrate how switching on the DNA mobility inside λ-capsid, by increasing the temperature to 37°C, facilitates the initiation of DNA ejection *in* *vitro*.

### Effect of intracapsid DNA mobility on the kinetics of initiation of DNA ejection

**Figure 5.**
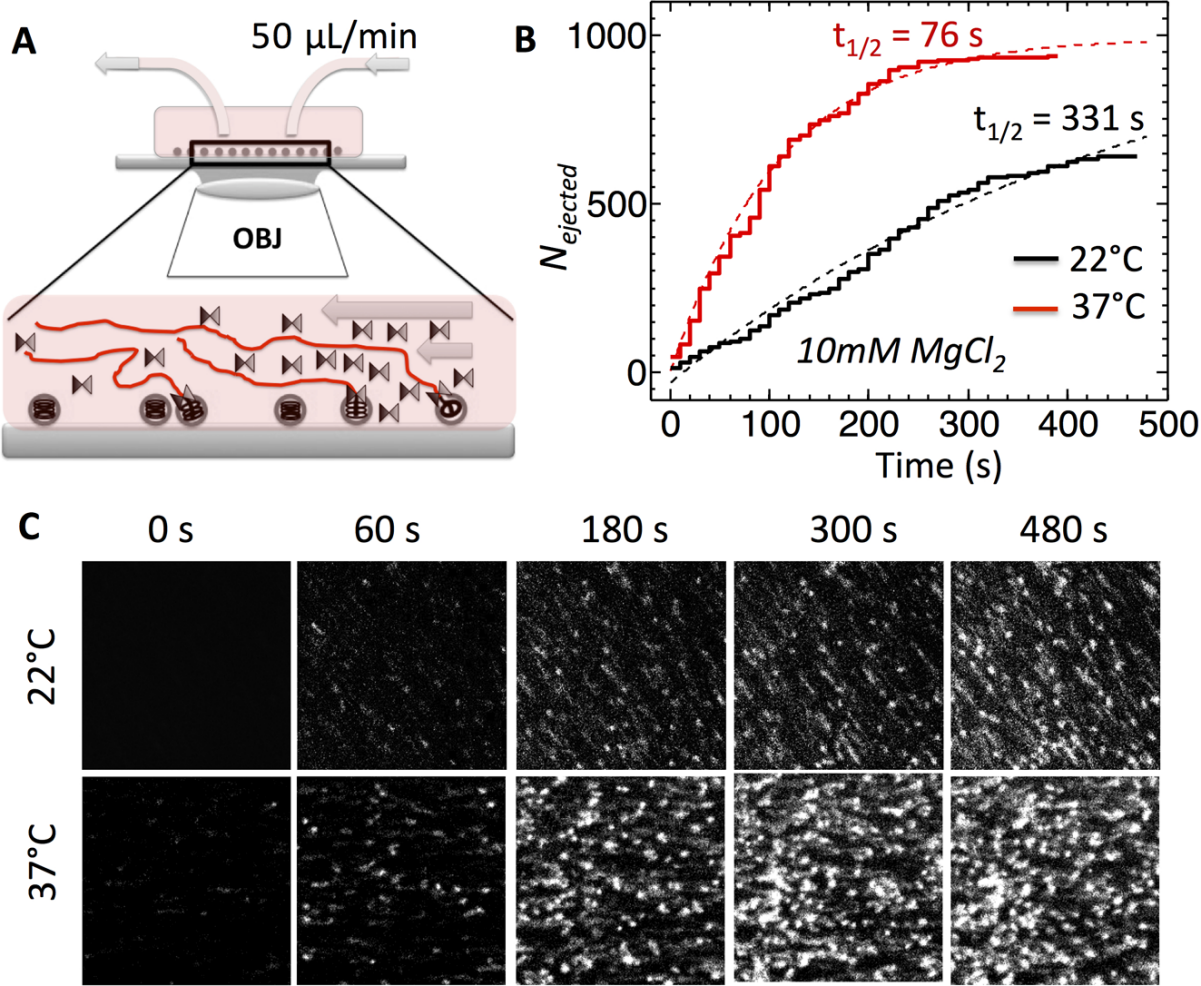
Single-molecule fluorescence measurements of the ensemble kinetics for DNA ejection from WT phage *λ* over time in 10 mM MgCl_2_ Tris-buffer at 22 and 37°C. YOYO dye shows particles with ejected DNA stretched in the flow. The ejection is triggered by LamB receptor addition at time 0 and flown continuously with YOYO in the flow chamber (**A**). The plots show the total number of ejections as a function of time at each temperature (**B**). The dotted lines are the single exponential fits of the ejection events assuming one-step kinetics. Half-time for the ejected *λ* particles is derived from the rate constants obtained from these fits. At 22°C *t_1/2_ =* 331 s, while at 37°C *t_1/2_ =* 76 s. To account for delay due to LamB delivery time to the flow chamber, the time is set to *t* = 0 when the first ejections are observed. The images in (**C**) are showing a cropped view (200 × 200 pixels) for the purpose of illustration. The original field of view used to digitally count the ejected particles (up to 1000 particles) was 512 × 512 pixels.

Using single-molecule fluorescence measurements, we measured the average ensemble kinetics of the number of phages which have ejected their DNA versus time once LamB has been added, see Figure 5. It is important to emphasize that the ejection time for individual phage particles is significantly shorter than the time frame for our measurements, which we confirmed with single-molecule fluorescence measurements and which was also verified in refs. (67,68). The LamB was used in excess (1:10 000 phage to LamB ratio) so that the initiation of DNA ejection was not limited by the LamB diffusion (this was experimentally confirmed).

We performed fluorescence measurements of DNA ejection using a spinning disk confocal microscope. Phage λ particles were adsorbed to a hydrophobically modified glass surface in a flow cell chamber. Fluorescent dye (YOYO^®^-1) is flown in together with LamB at time zero. The diffusion of YOYO into the capsid interior is strongly kinetically limited (69) (<1% of DNA-filled phage particles were stained within 10 min of incubation). However, once the DNA is ejected, YOYO instantly binds to it and indicates the number of phage particles that have ejected genome, appearing as fluorescent spots over time, Figure 5. Low flow rate was applied (50 μl/min) to avoid bleaching of the DNA-bound dye during the time of the measurements, which allows a continuous supply of the new dye and LamB molecules. The ejected DNA remains attached to the capsid, which was also observed in ref. (68). Furthermore, the ejected DNA immediately adheres to the modified glass surface and appears stretched in the flow, which helps with visualization of phages that have ejected their genomes. Figure 5 demonstrates that the ejection from all phage particles does not start simultaneously.

We measured population ejection kinetics in MgCl_2_ Tris-buffer for WT DNA length phage λ below and above the DNA transition temperature (T*∼33°C), at 22°C and at the physiological temperature of infection, 37°C. To quantify the difference in ejected phage population kinetics below and above the DNA transition temperature, we determined the total number of virions that ejected DNA versus time (see Figure 5B). A single exponential function, corresponding to a one-step kinetic process, was fitted for each dataset at 22 and 37°C, *a(1 − exp(−kt)) + b*, where *k* is the rate constant for ejection events (68). The half-time, *t_1/2_*, at which half of the phage population has ejected their genome is calculated from *t_1/2_ = ln2 / k*. As shown in Figure 5B, at 22°C *t_1/2_ =* 331 s, while at 37°C *t_1/2_ =* 76 s. Thus, after the DNA transition in the capsid has occurred, the half-time for the total number of ejected phage particles has dramatically decreased by ∼4.4 times.

These kinetics data illustrate that the intracapsid DNA mobility, regulated by the temperature and ionic conditions, strongly affects the ability of the virus to initiate its genome release, which is likely to affect the rate of viral replication *in* *vivo*. Interestingly, using plaque assays, we found that the average area of phage λ plaques formed on a fixed layer of *E. coli* cells during the same incubation time of 12 h has strong temperature dependence at temperatures above the intracapsid DNA transition temperature (T*∼33°C in MgCl_2_ Tris-buffer), see Supplementary Figure S4 in Supplementary Materials. Both phages and cells were re-suspended in 10 mM MgCl_2_ Tris-buffer (also used for the *in* *vitro* measurements above, see Supplementary Materials for a detailed method description). Remarkably, the plaque area is essentially unchanged between 30–35°C. However, it increases rapidly once the favorable temperature of infection is reached at T ≥ 37ºC and is almost doubled when the temperature is increased from 37 to 42°C. Likewise, many factors can be contributing to this behavior (70,71), but the temperature sensitive variation in the intracapsid DNA mobility could also potentially play a role.

## CONCLUSIONS

We found striking evidence that the packaged genome density in phage λ, corresponding to WT λ-DNA length (as opposed to the shorter λ-DNA length mutants), presents the most energetically and structurally optimized balance between genome mobility and its internal stress, both of which are required for efficient DNA ejection from the capsid. We show that DNA transition occurs once the critical DNA stress level in the capsid is reached. The genome stress is generated by interstrand repulsive interactions, packing defects and DNA bending stress, which are in turn controlled by the temperature and the ionic conditions of the surrounding solution. We discovered that at the most favorable external Mg^2+^-concentration for phage λ adsorption to *E. coli* and subsequent replication (∼10–20 mM Mg^2+^) (28), the DNA transition in the capsid occurs precisely at the physiologic temperature of infection (37°C). This suggests a remarkable evolutionary adaptation of the DNA transition mechanism in phage λ to the temperature and ionic environment of its host (i.e. *E. coli* in the human gut). Thus, DNA transition in the capsid is physiologically relevant, which suggests its importance for viral replication.

Both temperature and the surrounding conditions influence viral replication by ‘switching on’ the mobility of the encapsidated genome, which facilitates initiation of its rapid release into the cell. We also demonstrate that the DNA transition can be ‘switched off’ by varying the ionic conditions. Restricting intracapsid DNA mobility can have a virucidal effect (5,6), which is less prone to genetic mutations in the viral genome that can lead to development of drug resistance.

Various aspects of *genetic evolution* have been investigated in the field of virology (7). The *physical aspects* of viral evolution, however, are less understood. Recently, an intimate relationship was found between the physical and genetic evolution of dsDNA viruses (72). Specifically, it was shown that the physical limit of DNA length imposed by the capsid volume has led to gene overlap evolving as a mechanism for producing more proteins from the same genome length (72). This demonstrates how a *genetic mechanism* has evolved from a *physical* constraint by the capsid on the packaged genome density. Similarly with this finding, our data suggest that the genome packing density in phage λ is unique and highly conserved. This precise packing density not only determines the number of genes required for replication, but also creates an internal stress in the capsid required for the solid-to-fluid like DNA transition to occur at the temperature of infection. Such intracapsid DNA transition in λ facilitates initiation of DNA delivery from phage into a cell, and was shown to markedly increase the number of infectious viral particles once the transition temperature was reached (5). It is interesting to note that many of the well-studied dsDNA tailed bacteriophages (e.g. P22, T7, T2, T4, ϕ29, P2, P4) have similar DNA packing densities to that of phage λ, 44–48 bp/100 nm^3^ (73–75). This suggests that intra-capsid DNA transition behavior could regulate replication also in those viruses. Several eukaryotic dsDNA viruses (e.g. Herpesviruses) also have high DNA packing densities similar to those in phages. The DNA packing density in HSV-1 is slightly lower than in the WT DNA length phage λ (∼40 bp/100 nm^3^ versus ∼45 bp/100 nm^3^) and corresponds to the packing density in the 78% of WT λ-DNA length packaged in phage λ. We found that an analogous metastable DNA state exists inside the HSV-1 capsid, with solid-to-fluid like DNA transition occurring close to 37°C (6,9). This occurs when Mg^2+^ ions are removed from the Tris-Mg buffer through addition of excess EDTA. The resulting EDTA-Tris buffer used for HSV-1 suspension had the same composition and ionic strength as the buffer used to induce DNA transition in the 78% DNA length phage λ, studied immediately above. However, these low ionic strength conditions correspond to the ionic environment in the neuronal cell cytoplasm which is the host environment for HSV-1 (6). This suggests that the DNA transition mechanism regulating infectivity may be universal for many pressurized DNA viruses.

In conclusion, correlating structure and energetics of the encapsidated genome with the efficiency of viral replication provides a unique approach to explore the connection between *physical* and *genetic* aspects of viral evolution and creates novel opportunities for control of viral infections.

## SUPPLEMENTARY DATA

Supplementary Data are available at NAR Online.

SUPPLEMENTARY DATA
