# Supplementary material for: Ionic switch controls the DNA state in phage λ
Source: Nucleic Acids Res. 2015 Jun 19;43(13):6348–58. doi: 10.1093/nar/gkv611 (PMC4513876; doi:10.1093/nar/gkv611)
Supplement: SUPPLEMENTARY DATA [file supp_43_13_6348__index.html]

Ionic switch controls the DNA state in phage λ — SUPPLEMENTARY DATA 

# Ionic switch controls the DNA state in phage λ

## SUPPLEMENTARY DATA

- SUPPLEMENTARY DATA
