# Supplementary material for: Ionic switch controls the DNA state in phage λ
Source: Nucleic Acids Res. 2015 Jun 19;43(13):6348–58. doi: 10.1093/nar/gkv611 (PMC4513876; doi:10.1093/nar/gkv611)
Supplement: SUPPLEMENTARY DATA [file supp_gkv611_nar-01143-f-2015-File007.pdf]

Supplemental Materials

Ionic Switch Controls the DNA State in Phage λ

Dong Li[1, †], Ting Liu[1,†], Xiaobing Zuo[2], Tao Li[2], Xiangyun Qiu[3], Alex Evilevitch[1,4,*]

[1]Physics Department, Carnegie Mellon University, Pittsburgh, PA 15213, USA
[2]X-ray Science Division, Advanced Photon Source, Argonne National Laboratory, Argonne, IL 60439, USA
[3]Department of Physics, The George Washington University, Washington, DC 20052, USA
[4]Department of Biochemistry and Structural Biology, Lund University, SE-221 00 Lund, Sweden

† - authors contributed equally to this work
* - corresponding author (alexe@cmu.edu)

Figure S1. ITC titrations of phage λ to LamB and phage λ to buffer. Ionic conditions were 10 mM $MgCl_2$, 50 mM Tris-HCl (pH 7.4) at 37ºC. Differential power (μcal/s) is recorded versus time in seconds.

Figure S2. Cutaway views of cryo-EM reconstructions of phage λ containing different amounts of DNA (100% and 78% of the WT λ-DNA length). Spacing between the outermost layers of the DNA can be observed, while the DNA becomes more disordered closer to the center of the capsid. Owing to the icosahedral symmetry imposed during the reconstruction, concentrically packed DNA within the capsid becomes shells of density. The density maps are from our ref.(1).

Figure S3: Comparison of intermolecular force curves ($\log_{10}$ of PEG osmotic pressure, Π, versus interhelical DNA-DNA spacing, D_{int}) measured for helices in 10 mM $MgCl_2$ Tris-buffer at 5, 20 and 50°C.

Figure S4: The averaged plaque area is plotted as a function of incubation temperature. At least 300 plaques were analyzed for each sample. Error bars show the standard deviation. Plaques were measured after 12 hr for all samples. The plaque area is associated with the rate of infection spread of phage λ at each temperature.

Materials and Methods:

Bacteriophage and LamB receptor

WT bacteriophage λ cI857, with a genome length of 48.5 kb was produced by thermal induction of lysogenic *E. coli* strain AE1 derived from S2773 strain. Phage purification details are described elsewhere(2). All phage samples were purified by CsCl equilibrium centrifugation and dialyzed from CsCl against $MgCl_2$ TM buffer (10 mM $MgCl_2$/50 mM Tris·HCl, pH 7.4) or $MgSO_4$ TM buffer (10 mM $MgSO_4$/50 mM Tris·HCl, pH 7.4). The final titer was ≈ 10^{12} virions/mL, which was determined by plaque assay. Empty phage particles were prepared by incubating WT phage with its extracted LamB receptor for 1h at 37°C. The details can be found elsewhere(3). The receptor was the LamB protein purified from pop 154, a strain of *E. coli* K12 in which the *LamB* gene has been transduced from *Shigella sonnei* 3070. The detailed preparation was previously described(3).

Isothermal titration calorimetry (ITC)

All calorimetric measurements were performed using the MicroCal iTC200 system manufactured by GE Healthcare, Life Sciences. The details of phage DNA ejection enthalpy measurements were previously described in ref.(4). The ejection enthalpy at each given temperature, $\Delta H_{ej}(T)$, was calculated using the following titrations: $\Delta H_{ej} = \Delta H_{phage\ to\ LamB} - \Delta H_{phage\ to\ buffer} - \Delta H_{buffer\ to\ LamB} + \Delta H_{buffer\ to\ buffer}$. $\Delta H_{phage\ to\ LamB}$ was measured by titrating 2.69 μL of λ particles at 5 - 6×10^{12} pfu/mL (8 - 10nM) concentration into 200 μL of LamB solution in the sample cell (reference cell was always filled with MilliQ water). LamB was at a concentration of 0.2 - 0.5 mg/mL (1.4 - 3.5 μM). The molar ratio between LamB

trimmers and phage λ particles in the sample cell was always kept above 10^4:1 (up to at least 3 titrations) to ensure that the maximum number of phage λ particles eject their genomes without delay (LamB concentration was tested for saturation). Both LamB and λ particles were in the same dilution buffer containing TM (10mM $MgCl_2$ or $MgSO_4$, as indicated, 50mM Tris, pH7.4) and 1% oPOE. $\Delta H_{phage\ to\ buffer}$ was measured when the same batch of λ particles was titrated into the dilution buffer. $\Delta H_{buffer\ to\ LamB}$ was measured when the dilution buffer was titrated into the LamB solutions described above. $\Delta H_{phage\ to\ buffer}$ and $\Delta H_{buffer\ to\ LamB}$ measurements were performed to quantify the dilution and mixing heat of particles and were subtracted from the final calculation of the DNA ejection enthalpy. The term $\Delta H_{buffer\ to\ buffer}$ must be added since it is subtracted twice in the equation above. Measurements were performed within the temperature range between 18°C - 42°C.

<u>SAXS</u>
Small angle X-ray scattering (SAXS) measurements were carried out at the 12-ID B station at the Advanced Photon Source (APS) at Argonne National Laboratory. A 12KeV X-ray beam was used to illuminate the sample with an overall q range from 0.006 to 0.850 $Å^{-1}$. Total of 120 μL of WT phage solution (~ 5×10^{13} pfu/mL) was injected into a flow-through glass capillary and the solution was oscillating during the SAXS measurement with a flow rate of 10 μL/s. 40 scans with 1 second X-ray exposure time were collected and averaged for each sample.

<u>X-ray measurement of DNA-DNA forces in condensed arrays</u>
The osmotic stress technique for measuring forces is described in ref.(5). Force measurements were carried out at the Laboratory of Physical and Structural Biology, Program in Physical Biology, National Institutes of Health. Ni-filtered Cu-Ka radiation from an UltraBright microfocus x-ray source from Oxford Instruments equipped with polycapillary focusing x-ray optics was used for the small angle x-ray scattering (SAXS) experiments. The primary beam was also collimated by a set of slits. After equilibration, samples were sealed with ~ 100 ml equilibrating salt-PEG solution in a sample cell and mounted into a temperature-controlled holder. The flight path between the sample and detector, ~ 16 cm, was helium filled. Typical exposure times were ~ 30 min. Further details are described elsewhere(6).

<u>Fluorescence measurements:</u> Purified phage λ at 10^{14} pfu/mL were dialyzed and diluted 5000 times in 10 mM $MgCl_2$ 50 mM Tris-buffer for imaging purposes. Diluted phages were incubated on modified glass cover slips for at least 10 minutes before imaging and then washed with the same dilution buffer. After incubation, phages were exposed to the YOYO®-1 dye (by life technologies) at 100nM for imaging. LamB receptor solubilized in 1% oPOE was continuously flown into the chamber with YOYO dye. Phage particles were then imaged using a Nikon 2000E2 microscope with a spinning disk confocal scan head (Yokagawa Industries, Tokyo Japan). Images were collected using a 488 nm laser at 60X 1.45NA objective and EMCCD camera (Photometrics Cascade 2) for a period of 10 to 15 minutes until no further ejection events were observed. Fluorescence images were processed using ImageJ (public domain Java image processing program, NIH).

<u>Plaque assay analysis:</u> Purified phages were dialyzed in $MgCl_2$ Tris-buffer with 50mM Tris, 10mM $MgCl_2$ at pH 7.4 overnight, then diluted to 10^4 pfu/mL in the same buffer. *E. coli* C600 cells were grown to an OD_{600} of 0.5 in LB medium supplemented with 2mg/mL thymine and

0.2% maltose. Cells were then spun down and re-suspended in $MgCl_2$ Tris-buffer. 100 μL diluted phage samples were mixed with 200 μL pre-chilled re-suspended C600 cells, this mixture was then spread on the LB agar plates. The plates were incubated at desired temperatures for 12hr and then plaque area was determined. At least 300 plaques were counted on each plate for the plaque area determination.

REFERENCES:

1. Lander, G.C., Johnson, J.E., Rau, D.C., Potter, C.S., Carragher, B. and Evilevitch, A. (2013) DNA bending-induced phase transition of encapsidated genome in phage lambda. *Nucleic acids research*, **41**, 4518-4524.
2. Evilevitch, A., Lavelle, L., Knobler, C.M., Raspaud, E. and Gelbart, W.M. (2003) Osmotic pressure inhibition of DNA ejection from phage. *Proceedings of the National Academy of Sciences of the United States of America*, **100**, 9292-9295.
3. Ivanovska, I., Wuite, G., Jonsson, B. and Evilevitch, A. (2007) Internal DNA pressure modifies stability of WT phage. *Proc Natl Acad Sci U S A*, **104**, 9603-9608.
4. Jeembaeva, M., Jonsson, B., Castelnovo, M. and Evilevitch, A. (2010) DNA heats up: energetics of genome ejection from phage revealed by isothermal titration calorimetry. *Journal of molecular biology*, **395**, 1079-1087.
5. Parsegian, V.A., Rand, R.P., Fuller, N.L. and Rau, D.C. (1986) Osmotic-Stress for the Direct Measurement of Intermolecular Forces. *Methods in enzymology*, **127**, 400-416.
6. DeRouchey, J., Hoover, B. and Rau, D.C. (2013) A comparison of DNA compaction by arginine and lysine peptides: a physical basis for arginine rich protamines. *Biochemistry*, **52**, 3000-3009.

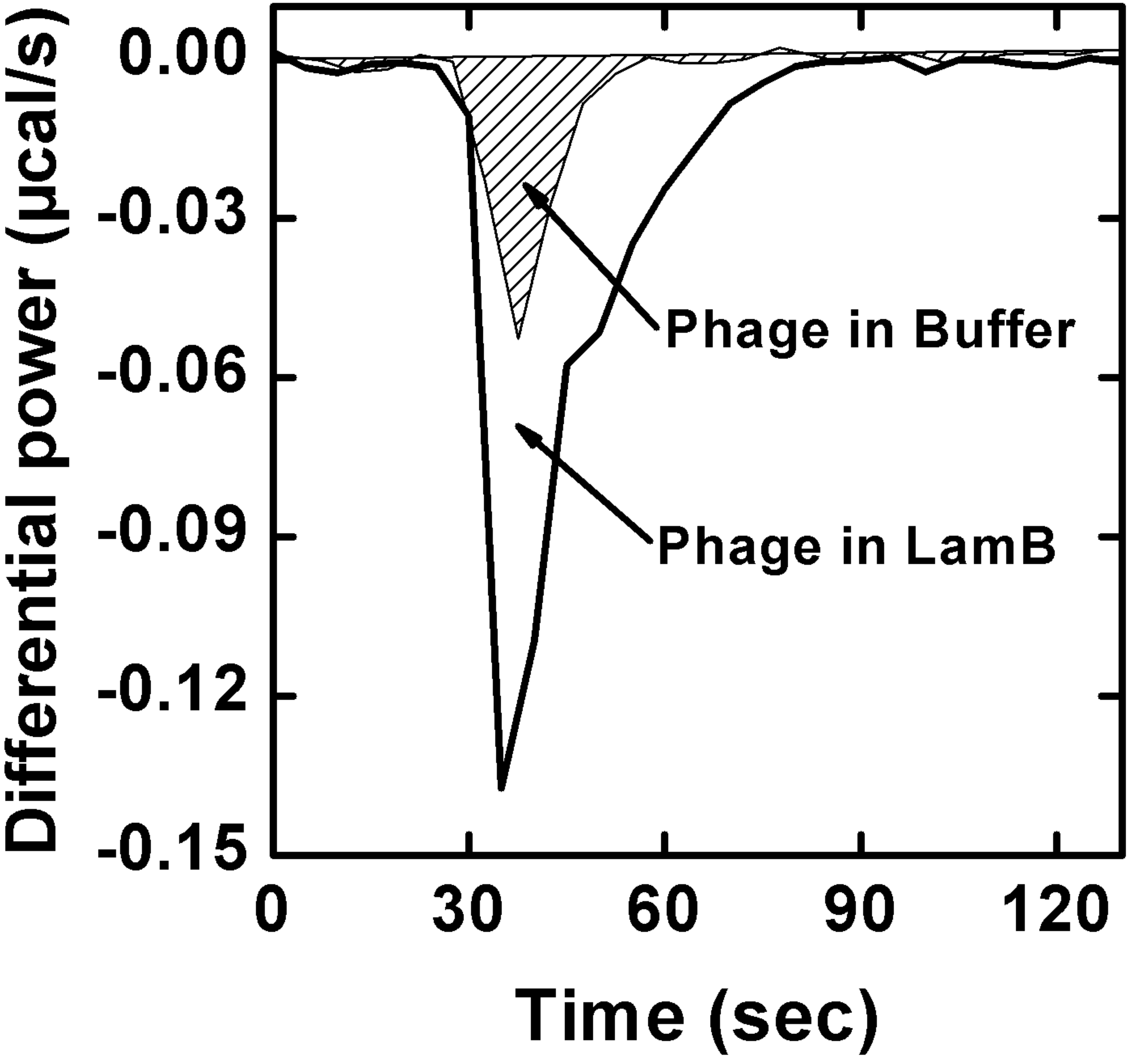

Differential power (µcal/s)
0.00
-0.03
-0.06
-0.09
-0.12
-0.15
0
30
60
90
120
Time (sec)
Phage in Buffer
Phage in LamB

WT DNA phage λ **78% of WT DNA phage λ**

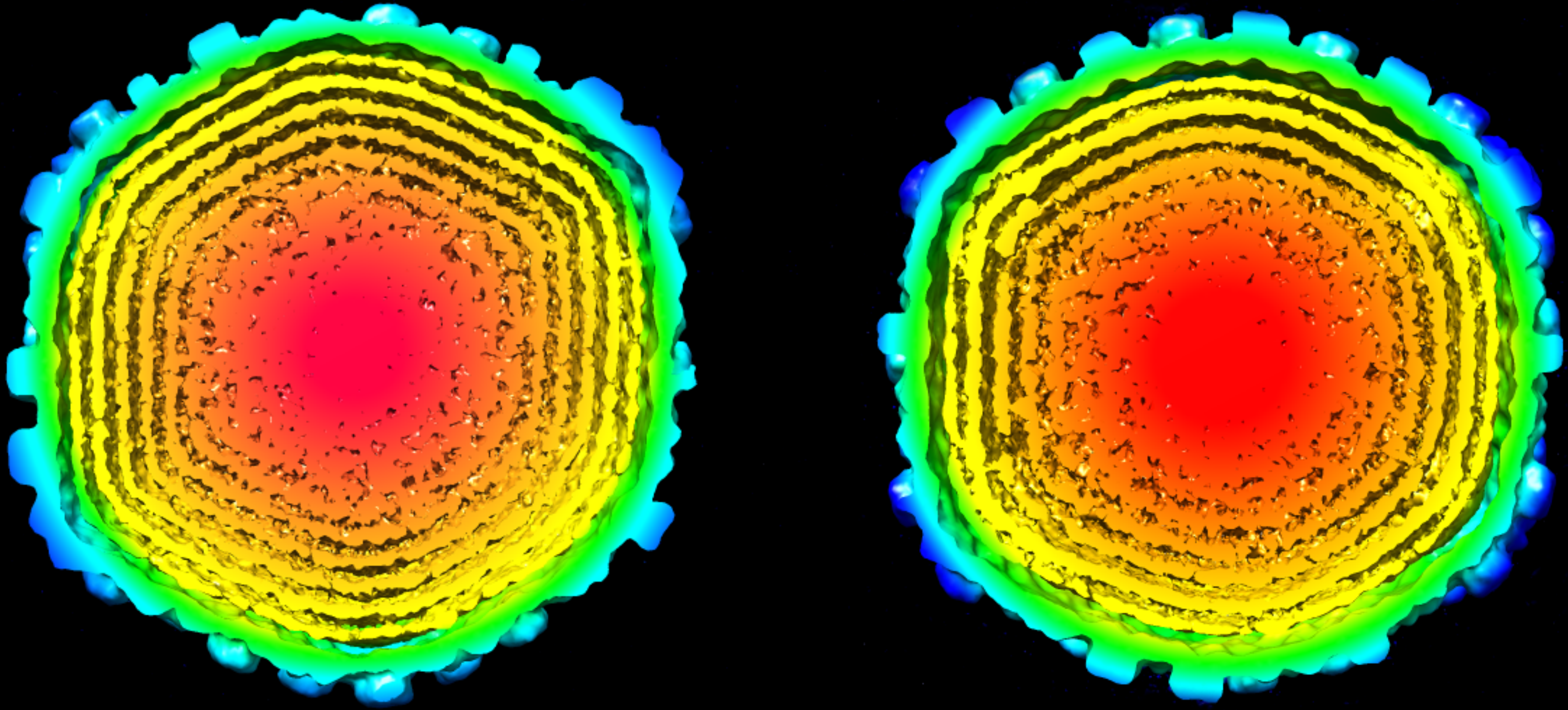

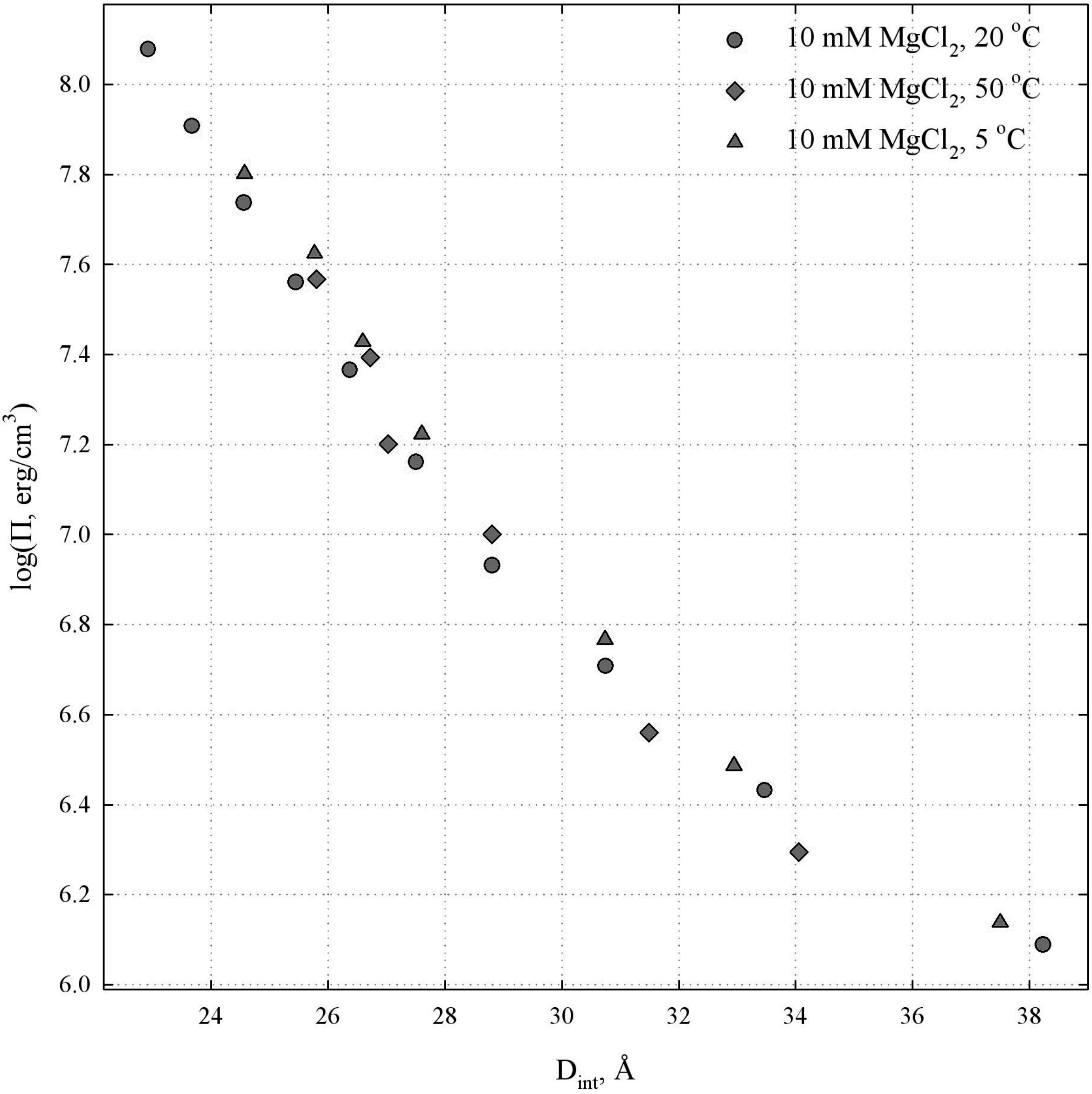

10 mM MgCl2, 20 oC
10 mM MgCl2, 50 oC
10 mM MgCl2, 5 oC
log(Π, erg/cm3)
8.0
7.8
7.6
7.4
7.2
7.0
6.8
6.6
6.4
6.2
6.0
24
26
28
30
32
34
36
38
Dint, Å

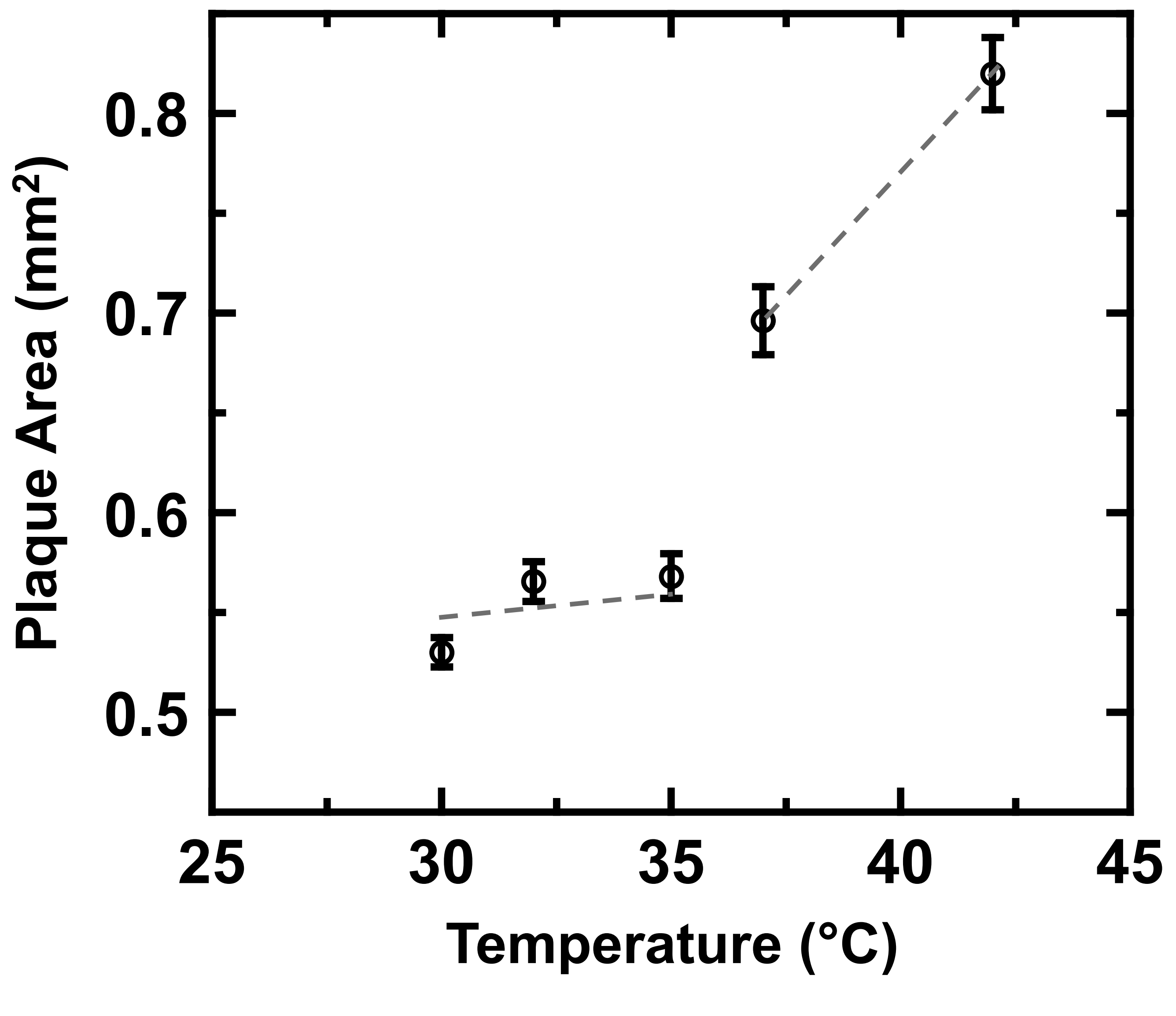
Plaque Area (mm²)
0.8
0.7
0.6
0.5
25
30
35
40
45
Temperature (°C)
